# Supplementary material for: Observation of a Correlation Between Internal friction and Urbach Energy in Amorphous Oxides Thin Films
Source: Sci Rep. 2020 Feb 3;10:1670. doi: 10.1038/s41598-020-58380-1 (PMC6997387; doi:10.1038/s41598-020-58380-1)
Supplement: Supplementary file 1 — Supplementary Information. [file 41598_2020_58380_MOESM1_ESM.pdf]

Supplementary information:

Observation of a Correlation Between Internal friction and Urbach Energy in Amorphous Oxides Thin Films

Alex Amato[1,*], Silvana Terreni[2], Massimo Granata[1], Christophe Michel[1], Benoit Sassolas[1], Laurent Pinard[1], Maurizio Canepa[2,3], and Gianpietro Cagnoli[4]

[1]Laboratoire des Matériaux Avancés, CNRS/IN2P3, F-69622 Villeurbanne (FR).
[2]OPTMATLAB, Dipartimento di Fisica, Università di Genova, Via Dodecaneso 33, 16146 Genova, Italy.
[3]INFN, Sezione di Genova, Via Dodecaneso 33, 16146 Genova, Italy.
[4]Université de Lyon, Université Claude Bernard Lyon 1, CNRS, Institut Lumière Matière, F-69622, VILLEURBANNE, France.
[*]a.amato@lma.in2p3.fr

ABSTRACT

This file includes a comparison of the Cody-Lorentz model (CL) with the point-by-point (pt. by pt.) fitting of the measured spectroscopic ellipsometry (SE) data, focusing on the Urbach tails region.

In figure 1, the Ψ, Δ data of Ti:Ta_2O_5 coating annealed at 500°C with fit curves are shown. Ti:Ta_2O_5 coating after the annealing at 500°C is the material currently adopted in the gravitational-wave detectors. For clarity, the comparison is presented for one incident angle only (60°). In figure 1(a),(b) fit with the CL model is presented. In figure 1(c),(d) the point-by-point analysis is presented; the thickness was setted at the same value found in the CL model. Looking at the residuals (the "Difference" panels) it is evident that the pt. by pt. analysis reproduces better Ψ, Δ data. This is related to the fact that the pt. by pt. fitting is able to reproduce all the nuances of data (including noise). The pt. by pt. analysis does not warrant the fulfilment of continuity conditions and Kramers-Kronig (KK) consistency though. On the other hand, the CL model allows to reproduce the Ψ, Δ data in the whole measured range respecting the continuity and KK consistency (we address the reader to a recent work[1], which discusses the limitation of KK consistency of CL model).

A close look to figure 1 shows that the CL model reproduces rather well the amplitude of the oscillations, related to the optical absorption properties of the coating. Looking at the residuals, the major differences between CL model and Ψ, Δ data are related to small discrepancies regarding the interference peaks positions.

In figure 2 we report a comparison between pt. by pt. and CL model fitting regarding the onset of the fundamental absorption edge, mostly relevant for the determination of Urbach energy parameter. In particular, we present the comparison of the annealed Ti:Ta_2O_5 coating and the as deposited Ta_2O_5 coating. The extinction coefficient and absorption α of annealed Ti:Ta_2O_5 are shown in figure 2(a) and 2(b), respectively. The extinction coefficient and α of as deposited Ta_2O_5 coating are shown in figure 2(c) and 2(d), respectively. The Urbach tails region is highlighted by the grey shadowing. The Urbach tails display a linear behaviour in logarithmic plot of α vs. E (figure 2(b),(d)).

The comparison shows that the CL model and the pt. by pt. curve present the same behaviour in the Urbach tails region.

We extrapolated the Urbach energy from the linear trend of the point-by-point curve, related to the Urbach tails and we compared the obtained values with those fitted using the CL model. The values of the Urbach energy extrapolated by the pt. by pt. analysis $E_U \sim 110$ meV and $E_U \sim 156$ meV are comparable to those obtained by the CL model $E_U = 108 \pm 5$ meV and $E_U = 164 \pm 8$ meV for the annealed Ti:Ta_2O_5 coating and not annealed Ta_2O_5 coating, respectively.

References

1. Larruquert, J. I. & de Marcos, L. V. R. *Thin Solid Films* **664**, 52 – 59, DOI: https://doi.org/10.1016/j.tsf.2018.08.010 (2018).

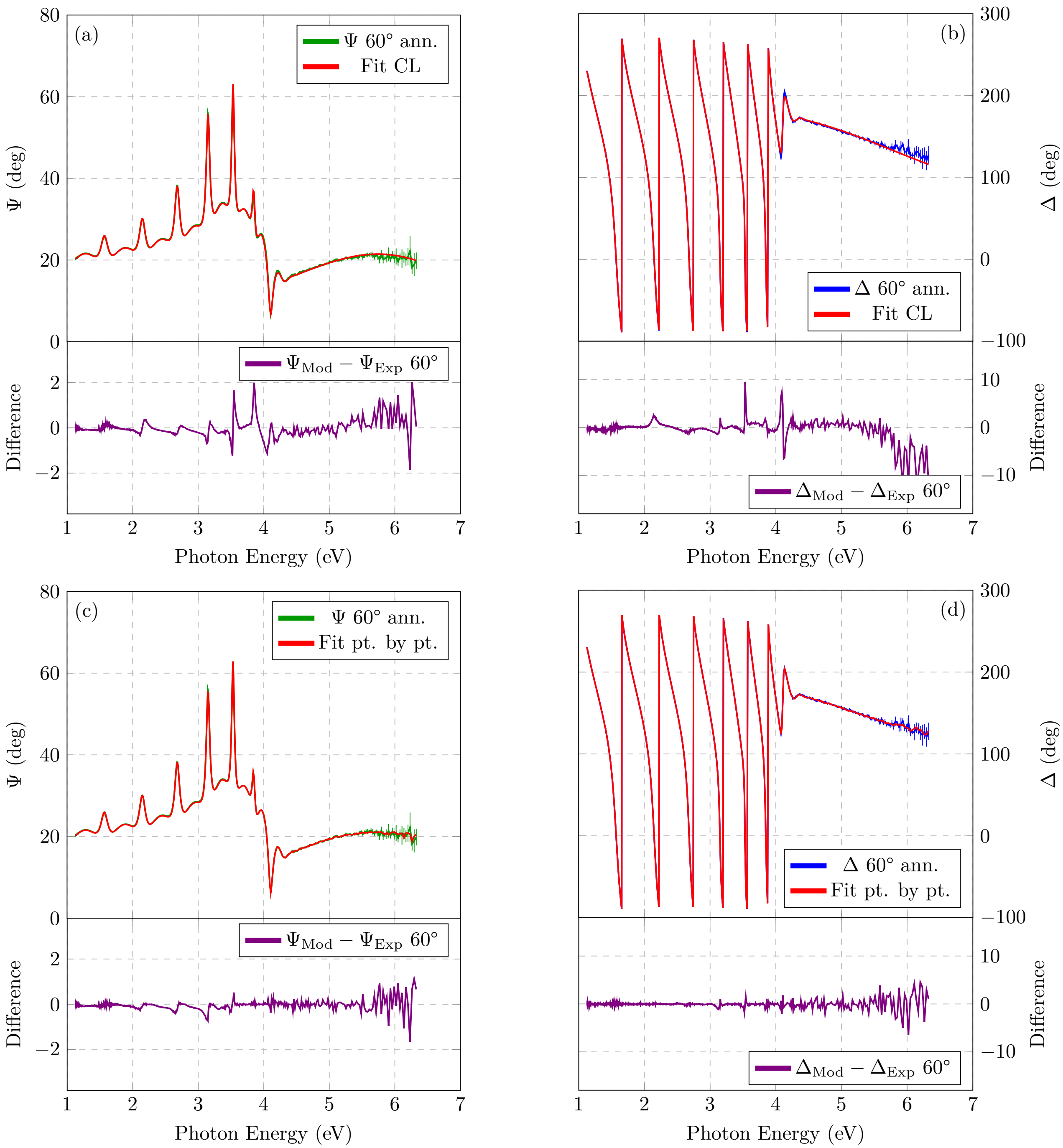

Figure 1. SE measurements acquired with an angle of incidence of 60° for Ti:Ta_2O_5 coating annealed at 500°C and fit (red curves). (a) and (b) Ψ and Δ data and fit curve obtained by the Cody-Lorentz model, respectively. (c) and (d) Ψ and Δ data and fit curve obtained by the point-by-point procedure, respectively. Under each panel the difference between measurement and fit, residual, is shown (violet).

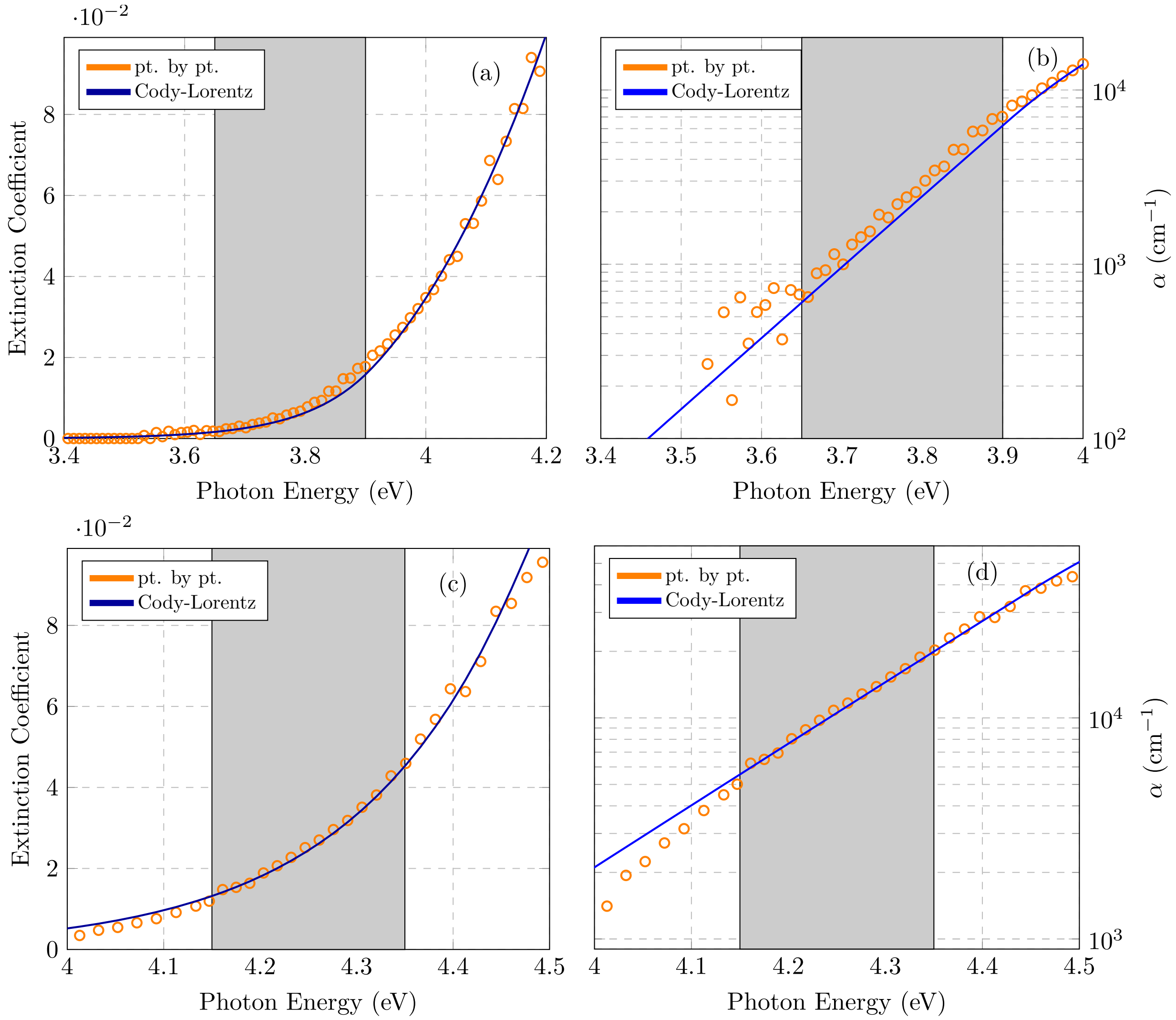

Figure 2. Comparison of extinction coefficient and absorption obtained by Cody-Lorentz (blue) and point-by-point fitting procedure (orange). (a),(b) curves related to Ti:Ta_2O_5 annealed coating. (c),(d) curves related to the Ta_2O_5 not annealed coating. The grey area highlights the Urbach tails region which is near the energy gap ($E_g = 3.6 \pm 0.1$ for Ti:Ta_2O_5 annealed coating and 4.1 ± 0.2 for Ta_2O_5 not annealed coating).
